# Technique for stretching a bent guidewire with a biopsy forceps in endoscopic ultrasound-guided hepaticogastrostomy

**DOI:** 10.1055/a-2491-4524

**Published:** 2024-12-17

**Authors:** Yoshitaka Nakai, Osamu Araki, Takeharu Nakamura, Kentaro Aoki, Yoshio Itokawa, Shigehiko Fujii

**Affiliations:** 1 38104 Department of Gastroenterology and Hepatology, Kyoto Katsura Hospital, Kyoto, Japan

Intraductal manipulation of the guidewire has been reported to be the most challenging step in endoscopic ultrasound-guided hepaticogastrostomy (EUS-HGS) 1 . Guidewire deviation not only results in an unsuccessful procedure, but can also cause bile leakage 2 . We hereby report a case in which the placed guidewire deviated during the EUS-HGS procedure, but we were able to recover it using biopsy forceps ( Video 1 ).

A rescue technique using a biopsy forceps to straighten a bent and deviated guidewire is performed during endoscopic ultrasound-guided hepaticogastrostomy.Video 1

Endoscopic retrograde cholangiopancreatography (ERCP) was performed on a 90-year-old woman with acute cholangitis and a history of distal gastrectomy (Billroth-II reconstruction) ( Fig. 1 **a, b** ). During insertion of the scope, a perforation was observed in the afferent loop, which was sutured with some clips. Because the intrahepatic bile duct was dilated ( Fig. 1 **b** ), we decided to perform EUS-HGS for drainage. The B2 bile duct was punctured from the stomach using a 19-gauge needle, and a 0.025-inch guidewire (Visiglide2; Olympus Inc., Tokyo, Japan) was placed into the common bile duct.

**Fig. 1:**
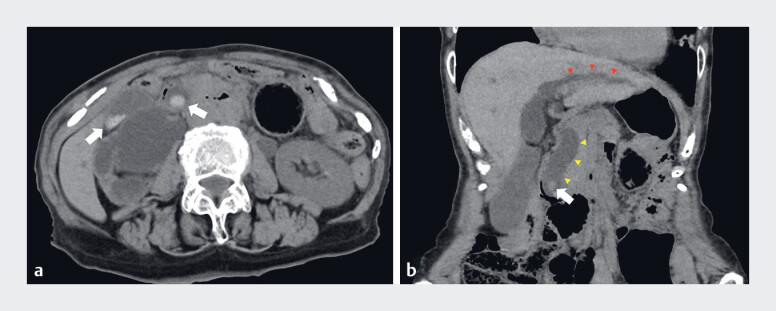
Abdominal computed tomography images showing: **a** choledocholithiasis, with multiple gallstones (white solid arrows); **b** dilatation of the common bile duct (yellow arrowheads) and intrahepatic bile ducts (red arrowheads).

During placement of a 6-Fr endoscopic nasobiliary drainage (ENBD) tube, the guidewire flexed in the stomach and was difficult to restraighten by guidewire and scope manipulation. The guidewire was therefore grasped with biopsy forceps (1C Biopsy Forceps, 1.8 mm; Micro-Tech Co., Ltd., Nanjing, China), and we were able to straighten and shorten it by simply pulling it out through the forceps channel ( Fig. 2 and Fig. 3 ). An ENBD was inserted up to the duodenum to complete the procedure. The subsequent course of the perforation and acute cholangitis was uneventful. Stone removal was difficult owing to stenosis of the afferent loop, so the patient was discharged about 2 weeks after drainage with a plastic stent.

**Fig. 2:**
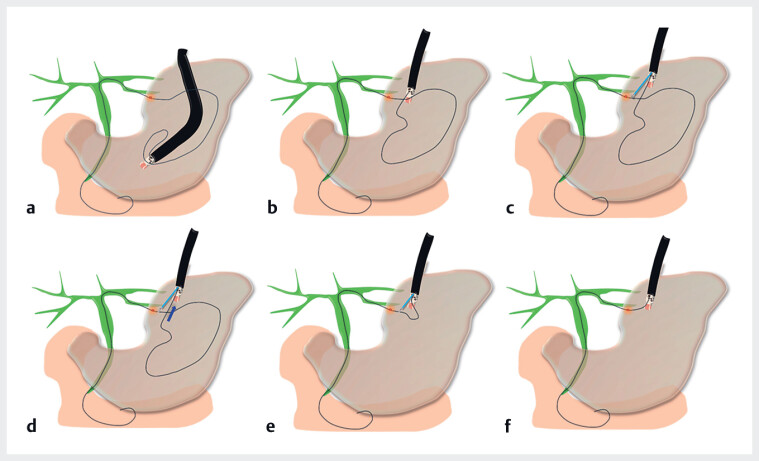
Schematic showing: **a, b** the scope guided to the puncture site; **c, d** the guidewire grasped by biopsy forceps; **e, f** the guidewire pulled out through the forceps channel and stretched.

**Fig. 3:**
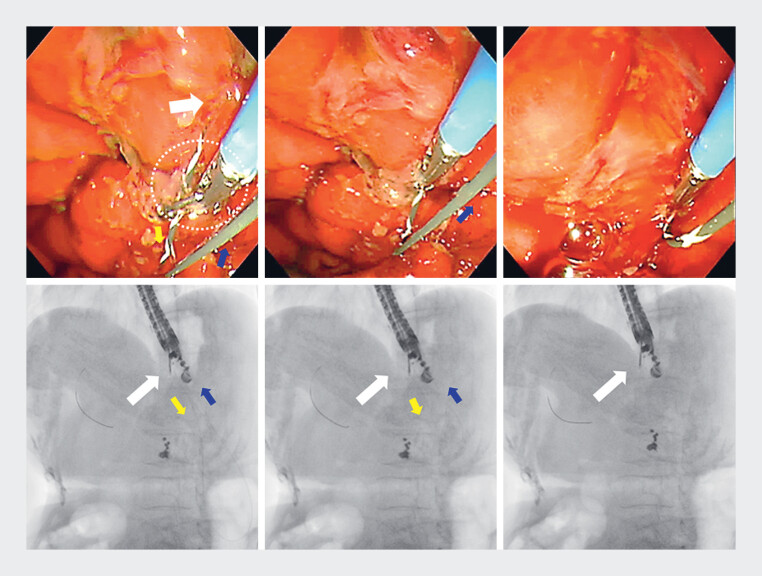
Endoscopic and fluoroscopic images showing the technique for stretching the guidewire by grasping with a biopsy forceps (white dotted circle). White arrow, puncture site; yellow arrow, anal side guidewire; blue arrow, oral side guidewire.

The use of the double-guidewire technique for safe guidewire use has also been reported 3 . Our technique may be simple and useful, not only in EUS-HGS, but also in ERCP and other EUS-related interventions.

Endoscopy_UCTN_Code_CPL_1AL_2AD

## Notes

Endoscopy E-Videos https://eref.thieme.de/e-videos
*E-Videos* is an open access online section of the journal *Endoscopy* , reporting on interesting cases and new techniques in gastroenterological endoscopy. All papers include a high-quality video and are published with a Creative Commons CC-BY license. Endoscopy E-Videos qualify for HINARI discounts and waivers and eligibility is automatically checked during the submission process. We grant 100% waivers to articles whose corresponding authors are based in Group A countries and 50% waivers to those who are based in Group B countries as classified by Research4Life (see: https://www.research4life.org/access/eligibility/ ). This section has its own submission website at https://mc.manuscriptcentral.com/e-videos .
